# Race and ethnicity moderate the associations between lifetime psychedelic use (MDMA and psilocybin) and psychological distress and suicidality

**DOI:** 10.1038/s41598-022-18645-3

**Published:** 2022-10-10

**Authors:** Grant M. Jones, Matthew K. Nock

**Affiliations:** Department of Psychology, Harvard University, 33 Kirkland St, Cambridge, MA 02138 USA

**Keywords:** Psychology, Human behaviour

## Abstract

Psychedelic compounds have been linked to salutary mental health outcomes in both naturalistic and clinical settings; however, current research on psychedelics suffers from a lack of inclusion and focus on racial and ethnic minorities. Thus, the goal of our study was to assess whether race and ethnicity moderate the associations that naturalistic lifetime MDMA (3,4-Methylenedioxymethamphetamine) use and psilocybin use share with past month psychological distress and past year suicidality (ideation and planning). Using data from the National Survey on Drug Use and Health (NSDUH) (2008–2019) (*N* = 484,732), we conducted survey-weighted multivariable logistic regression to conduct interaction tests and to assess the associations that MDMA use and psilocybin use share with the aforementioned outcomes for each racial and ethnic group. Race and ethnicity significantly moderated the associations between MDMA and psilocybin use and psychological distress and suicidality. For White participants, MDMA and psilocybin use conferred lowered odds of all distress and suicidality outcomes. For racial and ethnic minority participants, the associations between psychedelic use and suicidality were far fewer. These findings invite further research into the impact of race, ethnicity, and other identity factors (e.g., socioeconomic status, sexual/gender minority status) on the effects of psychedelic substances.

## Introduction

### MDMA and psilocybin as therapeutic agents

MDMA (3,4-Methylenedioxymethamphetamine) and psilocybin are psychedelic compounds that are receiving increased attention within clinical research for their potentially powerful therapeutic effects. MDMA (also colloquially referred to as “molly”) is a synthetic compound that is known to increase feelings of empathy and social connection and can facilitate the processing of challenging emotions. In naturalistic contexts, this compound is also referred to as “ecstasy” due to its euphoric effects. Psilocybin is a compound that elicits profound perceptual shifts and mystical-type experiences (e.g., feelings of unity and oneness with the universe, highly positive mood states, transcendence of time and space, etc.) that can have deep personal significance^1,2^. In contrast to MDMA, psilocybin is a naturally occurring classic psychedelic compound, a category of substances that are defined by their shared subjective effects as well as their serotonin 2A receptor agonist activity. Furthermore, psilocybin and other naturally occurring classic psychedelics like peyote (a psychoactive cactus native to North America), huachuma/San Pedro (a psychoactive cactus native to South America), mescaline (the primary psychoactive compound in peyote and huachuma/San Pedro), ayahuasca (a South American psychoactive plant brew), and dimethyltryptamine (DMT) (a psychoactive compound found in ayahuasca) have been ingested by Indigenous cultures for millennia for healing rituals and worship^3^.

Many clinical trials have demonstrated MDMA and psilocybin to hold therapeutic promise^4–10^. As a corollary of strong results in these clinical trials, the FDA has declared MDMA and psilocybin to be “Breakthrough Therapies” for treatment-resistant PTSD and severe depression, respectively. Additionally, other trials have suggested MDMA to be useful for other conditions, such as social anxiety^11^ and anxiety associated with end-of-life illnesses^12^; psilocybin has also demonstrated promise for treating substance use disorders as well^13,14^. Most recently, a 2022 meta-analysis conducted by Zeifman et al.^15^ analyzed the results of seven psychedelic clinical trials (five of which used psilocybin-assisted psychotherapy) and found large reductions in suicidality as well. Although the research is still in its early stages, existing research strongly reinforces the potential of MDMA and psilocybin as therapeutic agents.

### Race and ethnicity as a critical limitation within current psychedelic research

Despite the demonstrated potential of these compounds within psychedelic research, there is a lack of focus on how race (social categories created by colonialism that are loosely related to the ancestral backgrounds, histories, and physical characteristics of a given population) and ethnicity (social categories related to the shared cultural factors of a given population) may impact the effects of psychedelics, as most participants in psychedelic trials are White. A review featuring 17 foundational psychedelic studies conducted from 2000 to 2017 found that only 4.1% of all participants were of Black or Hispanic descent whereas 82.3% of participants were White^16^. More recent trials are also severely racially homogenous. A randomized controlled trial of psilocybin for depression conducted by Davis et al. (2020)^17^ notes this homogeneity to be a limitation of their study; additionally, another recent trial published by Carhart-Harris et al. (2021)^18^ comparing psilocybin to escitalopram for treating depression was 93% White. The homogeneity of existing trials sharply limits the generalizability of existing findings related to MDMA and psilocybin to minority populations.

Amidst this critical need for further research on MDMA and psilocybin for racial and ethnic minorities, there are a limited number of empirical investigations into use of these compounds in racial and ethnic minority populations. First, Williams et al. (2020)^19^ recruited 313 Black, Indigenous, and People of Color (BIPOC) participants and examined the link between naturalistic use of MDMA, psilocybin, and LSD (lysergic acid diethylamide—a classic psychedelic substance synthesized from the ergot fungus) and mental health in these populations. Overall, these researchers found that use of these substances promoted significant reductions in symptoms of racial trauma, depression, anxiety, and stress. Second, Davis et al. (2021)^20^ conducted follow up work and found that psychological flexibility mediated the associations between psychedelic use and reductions in racial trauma.

Despite the essential contributions these studies make to the scientific literature, these investigations also have limitations as well. First, these studies recruited only individuals who endorsed positive benefits of MDMA, psilocybin, and LSD on symptoms of racial trauma, biasing the participant pool and the results. Furthermore, these studies asked participants to retrospectively rate changes they experienced in their racial trauma and mental health symptoms following their use of these compounds, introducing the potential for recall bias. Thus, there is a need for more investigations into the potential salutary impact of psychedelics for diverse populations, as well as a need to address limitations within the scant existing research.

### Population-based survey approach

A population-based survey approach can allow us to address the aforementioned limitations of the two existing investigations into psychedelic use in racial and ethnic minority populations. In light of the participant pool bias of the previous studies, a population-based survey allows us access to participants with diverse substance use experiences and mental health histories. Additionally, in light of the retrospective bias limitations of Williams et al. (2020)^19^ and Davis et al. (2021)^20^, population-based survey approaches do not rely upon individuals to provide retrospective accounts of changes in their mental health outcomes following substance use. Although one cannot use population-based surveys to infer causality, this approach can nevertheless enable one of the most robust and nuanced investigations into the link between psychedelic use and minority mental health conducted to date.

Seminal research findings on psychedelics using population-based survey approaches have largely focused on outcomes related to psychological distress and suicidality; these outcomes will thus guide the focus of our study. Hendricks et al. (2015)^21^ used data from The National Survey on Drug Use and Health (NSDUH) (2008–2012) and found that lifetime use of classic psychedelics (psilocybin, LSD, mescaline, peyote, DMT, ayahuasca) was associated with lowered odds of psychological distress and suicidality. Sexton et al. (2020)^22^ conducted more granular analyses using a larger NSDUH dataset (2008–2017) and found that, of all classic psychedelics, classic tryptamines in particular (e.g., psilocybin, DMT, ayahuasca) conferred lowered odds of past month psychological distress and past year suicidal ideation. In one of the most recent and comprehensive analyses using the NSDUH survey, our research team analyzed all available NSDUH data from 2008 to 2019 to examine the relationships that specific psychedelics share with psychological distress and suicidality^23^. Overall, we found that lifetime MDMA use and psilocybin use in particular lowered odds of the aforementioned outcomes; additionally, to our knowledge, this study represents one of the first that specifically links MDMA use to lowered odds of general psychological distress and suicidality as well.

Thus, building on this latest research, here we explore how race and ethnicity moderate the associations between MDMA use and psilocybin use and psychological distress and suicidality.

## Method

Data for this project are from the National Survey on Drug Use and Health (NSDUH) (2008–2019), a yearly survey that collects data on mental health outcomes and substance use patterns within a nationally representative sample of the United States population (total unweighted *N* = 484,732). This study was exempt from review from the Harvard University IRB as these data are publicly available and all methods were carried out in accordance with the relevant guidelines and regulations.

### Independent variables/covariates

The independent variables within this study assess lifetime use (yes/no) of MDMA and psilocybin. In line with previous population-based research on psychedelics, we controlled for the following factors: sex (male or female), age (18–25, 26–34, 35–49, 50 or older), educational attainment (less than high school, some high school or high school graduate, some college or above), self-reported engagement in risky behavior (never, seldom, sometimes, or always), annual household income (less than $20,000, $20,000–$49,999, $50,000–$74,999, $75,000 or more), marital status (married, divorced/separated, widowed, or never married), survey year (2008–2019), and lifetime use of various substances (other classic psychedelics [LSD, peyote, and mescaline], other illegal substances [cocaine, heroin, PCP, inhalants] and other commonly misused legal/medicinal substances [pain relievers, tranquilizers, stimulants, sedatives, and marijuana]).

### Dependent variables

Within our analyses, we utilized three dependent variables (yes/no) that assessed psychological distress and suicidality; additionally, we selected these variables as prior research indicated that lifetime use of MDMA or psilocybin confers lowered odds of these metrics as well^23^.

#### Past month psychological distress

Past month psychological distress is a variable in the NSDUH survey that is based on six domains from the K6 screening instrument for non-specific psychological distress: feeling nervous, feeling hopeless, feeling restless or fidgety, feeling so sad or depressed that nothing could cheer you up, feeling that everything was an effort, and feeling down on yourself, no good, or worthless^24^. Individuals responded with a score of 0 (“none of the time”) to 4 (“all of the time”) for each domain, and individuals who scored 13 or greater (out of 24) were coded positively for past month serious psychological distress. This cut-off point was chosen in the NSDUH survey as scores above this threshold correlate with increased likelihood of serious mental illness^25^.

#### Past year suicidal thinking and planning

We include two suicidality variables: past year suicidal ideation (“At any time in the past 12 months…did you seriously think about trying to kill yourself?”) and past year suicidal planning (“During the past 12 months, did you make any plans to kill yourself?”). Although the NSDUH also includes a past year suicide attempt variable, we did not include this variable in our study as our prior work did not find any protective associations between psychedelic use and suicide attempts^23^.

### Putative moderator

#### Race and ethnicity

We created and used a three-level race and ethnicity variable (reduced from the seven-level race and ethnicity variable included in the NSDUH) to lower the number of interaction tests and reduce the likelihood of a type 1 error (false positive). The three levels were: Non-Hispanic White (*N* = 298,383), Non-Hispanic Racial or Ethnic Minority (*N* = 106,292), and Hispanic (*N* = 80,057). The Non-Hispanic Racial or Ethnic Minority category included the following racial and ethnic groups: Non-Hispanic Black (*N* = 60,714), Non-Hispanic Native American/Alaska Native (*N* = 7,161), Non-Hispanic Native Hawaiian/Pacific Islander (*N* = 2,470), Non-Hispanic Asian (*N* = 21,105), and Non-Hispanic Multiracial individuals (*N* = 14,842).

### Data analyses

We used the ‘Survey’ package^26^ in R version 4.1.2 to conduct our analyses, allowing us to incorporate the complex survey design and the sampling weights from the NSDUH into our study.

For Step 1, we used survey-weighted multivariable logistic regression to test whether race and ethnicity significantly moderated any associations between MDMA use and psilocybin use and the dependent variables. In interpreting these models, we were not concerned with the specific beta values yielded by these models but simply whether these tests were significant, as they would pave the way for the next step of our analyses.

For Step 2, for models in which there were any significant interactions between race and ethnicity and MDMA use or psilocybin use, we would subsequently divide our sample by race and ethnicity and conduct additional multivariable logistic regression models to generate adjusted odds ratios (aORs) and finally assess the relationships that MDMA use and psilocybin use share with psychological distress and suicidality for each identity group (White, Black, Native American/Alaska Native, Native Hawaiian/Pacific Islander, Asian, Multiracial, Hispanic). This approach allowed us to incorporate the unique substance use patterns and demographic profiles of each racial and ethnic group into our analyses.

In Step 2, MDMA and psilocybin use serve as the primary independent variables and lifetime use of all other substances and demographic factors serve as covariates. Additionally, for these analyses, we combined the Native American/Alaska Native and Native Hawaiian/Pacific Islander participants into one “Indigenous” category, as the Native Hawaiian/Pacific Islander group was too small for us to conduct survey-weighted regression models with participants from this population alone.

## Results

### Demographics and prevalence of psychedelic use, distress, and suicidality

The demographics of our sample as well as the prevalence of psychedelic use, distress, and suicidality are presented in Table 1. Rates of MDMA use and psilocybin use were higher among White participants than they were among racial and ethnic minority participants, whereas rates of psychological distress and suicidality were roughly similar for White and minority participants.

**Table 1:** Sample demographics and prevalence of psychedelic use, past month psychological distress, and past year suicidality, stratified by race and ethnicity.

Characteristic	Non-Hispanic White (weighted %)	Non-Hispanic racial or ethnic minority (weighted %)	Hispanic (weighted %)	p-value^1^
Lifetime MDMA use	7.9	5.2	6.0	< 0.001
Lifetime psilocybin use	12	3.4	5.2	< 0.001
**Marital status**				< 0.001
Married	57	42	49	
Widowed	6.7	5.3	3.4	
Divorced or separated	14	14	13	
Never married	23	39	35	
**Yearly household income**				< 0.001
< $20,000	14	26	24	
$20,000–$49,999	29	32	40	
$50,000–$74,999	18	15	14	
$75,000+	40	28	21	
**Educational attainment**				< 0.001
Less than HS	2.1	2.8	16	
Some HS or HS Grad	35	39	44	
Some college or above	63	58	40	
Past month distress	5.2	5.5	5.6	< 0.001
Past Yr. suicidal ideation	4.3	3.7	3.4	< 0.001
Past Yr. suicidal planning	1.2	1.2	1.0	< 0.001
Past Yr. suicide attempt	0.5	0.7	0.6	< 0.001

^1^Chi-squared test with Rao & Scott's second-order correction.

### Interaction tests

The interactions between MDMA and psilocybin use and race and ethnicity for predicting past month psychological distress, past year suicidal ideation, and past year suicidal planning are presented in Table 2. An “N/A” value indicates that we did not test for interaction as our previous research indicated that there was not an association between MDMA or psilocybin and the specific outcome of interest^23^. Overall, the “Non-Hispanic Racial or Ethnic Minority” category interacted significantly with psilocybin use for predicting past year suicidal ideation, and with MDMA use for predicting past year suicidal ideation and planning. “Hispanic” identity interacted with psilocybin use for predicting past month psychological distress and past year suicidal ideation, and with MDMA use for predicting past year suicidal ideation and planning. Thus, we proceeded with dividing our sample by race and ethnicity and testing the associations between the aforementioned substances and all distress and suicidality outcomes.

**Table 2:** Interactions between race and ethnicity and MDMA use and psilocybin use for predicting past month psychological distress, past year suicidal ideation (S.I), and past year suicidal planning (S.P).

	Past Mo. distress	Past Yr. S.I	Past Yr. S.P
Interaction test	beta (95% CI)^1^	beta (95% CI)	beta (95% CI)
**Race/ethnicity × MDMA**			
Non-Hispanic racial or ethnic minority	N/A^a^	0.45*** (0.30, 0.59)	0.41** (0.15, 0.67)
Hispanic	N/A	0.38*** (0.24, 0.53)	0.37** (0.10, 0.64)
**Race/ethnicity × Psilocybin**			
Non-Hispanic racial or ethnic minority	0.10 (−0.06, 0.27)	0.40*** (0.21, 0.59)	N/A
Hispanic	0.20* (0.03, 0.37)	0.41*** (0.22, 0.59)	N/A

All aforementioned demographic factors and lifetime use variables are included as covariates.

^a^An N/A value indicates we did not test for an interaction as our prior research (Jones and Nock (2022)^23^) indicated that there was not an association between MDMA or psilocybin and the specific outcome of interest.

^1^*p < 0.05; **p < 0.01; ***p < 0.001; CI = confidence interval.

### Associations between MDMA use and psilocybin use and psychological distress and suicidality, stratified by race and ethnicity

The associations of MDMA and psilocybin use with all three relevant outcomes, divided by race and ethnicity, are presented in Table 3. For White participants, lifetime MDMA use conferred lowered odds of all three outcomes: past month psychological distress (aOR: 0.89), past year suicidal ideation (aOR: 0.85), and past year suicidal planning (aOR: 0.82); additionally, psilocybin use conferred lowered odds of past month psychological distress (aOR: 0.80) and past year suicidal ideation (aOR: 0.90). For Hispanic participants, MDMA use predicted lowered odds of past year suicidal ideation (aOR: 0.81) and psilocybin use predicted lowered odds of past month psychological distress (aOR: 0.61). For Asian participants, only psilocybin use predicted lowered odds of past month psychological distress (aOR: 0.34). For Black, Indigenous, and Multiracial participants, MDMA and psilocybin use were not associated with lower odds of any outcomes.

**Table 3:** Associations between lifetime MDMA use and psilocybin use and past month psychological distress, past year suicidal ideation (S.I.), and past year suicidal planning (S.P), by race and ethnicity.

Group	Lifetime use	Past Mo. distress	Past Yr. S.I	Past Yr. S.P
		aOR (95% CI)^1^	aOR (95% CI)	aOR (95% CI)
White	MDMA	0.89** (0.82, 0.96)	0.85*** (0.78, 0.93)	0.82* (0.70, 0.96)
	Psilocybin	0.80*** (0.75, 0.87)	0.90* (0.82, 0.98)	0.90 (0.76, 1.07)
Black	MDMA	1.12 (0.91, 1.38)	1.07 (0.87, 1.32)	1.13 (0.79, 1.60)
	Psilocybin	0.96 (0.72, 1.28)	1.03 (0.74, 1.44)	1.21 (0.73, 2.01)
Indigenous	MDMA	1.28 (0.75, 2.19)	1.24 (0.74, 2.07)	1.27 (0.55, 2.97)
	Psilocybin	1.24 (0.69, 2.20)	0.80 (0.45, 1.44)	0.93 (0.44, 1.97)
Asian	MDMA	0.95 (0.57, 1.56)	0.93 (0.63, 1.39)	0.64 (0.34, 1.18)
	Psilocybin	0.34** (0.18, 0.64)	1.04 (0.52, 2.09)	0.55 (0.24, 1.24)
Multiracial	MDMA	0.83 (0.59, 1.17)	1.23 (0.93, 1.61)	1.07 (0.69, 1.67)
	Psilocybin	0.99 (0.64, 1.54)	1.13 (0.78, 1.62)	0.90 (0.50, 1.65)
Hispanic	MDMA	0.99 (0.82, 1.20)	0.81* (0.65, 0.99)	0.85 (0.61, 1.19)
	Psilocybin	0.61*** (0.48, 0.79)	0.92 (0.74, 1.14)	0.83 (0.58, 1.18)

Significant values that indicate lowered odds of distress or suicidality are in bold.

All aforementioned demographic factors and lifetime use variables are included as covariates.

^1^*p < 0.05; **p < 0.01; ***p < 0.001; aOR, adjusted odds ratio; CI, confidence interval.

### Exploratory analyses—associations between other classic psychedelics (LSD, peyote, mescaline) and psychological distress and suicidality, stratified by race and ethnicity

We also conducted additional survey-weighted multivariable logistic regression models, stratified by race and ethnicity, to examine whether other classic psychedelics (LSD, peyote, and mescaline) might confer lowered odds of psychological distress and suicidality. We conducted these exploratory analyses as they could reveal additional protective associations between psychedelic use and mental health outcomes for racial and ethnic minorities. The results of these analyses are presented in Supplemental Table 1. Notably, lifetime peyote use conferred significantly lowered odds of past month psychological distress for Indigenous participants (aOR: 0.55). Additionally, lifetime mescaline use yielded lowered odds of past year suicidal ideation for Multiracial individuals (aOR: 0.57). No other psychedelics were associated with lowered odds of distress and suicidality for racial and ethnic minorities in these analyses.

## Discussion

This study tested whether race and ethnicity moderate the associations that lifetime MDMA use and psilocybin use share with psychological distress and suicidality. Overall, we found significant interactions between race and ethnicity and MDMA use and psilocybin use across all relevant distress and suicidality outcomes. In examining the associations of MDMA use and psilocybin use with distress and suicidality by race and ethnicity, we see starkly different relationships across identity groups. For White participants, lifetime MDMA use and psilocybin use conferred lowered odds of all outcomes of interest related to psychological distress and suicidality. For Hispanic and Asian, participants, however, there were fewer of such relationships and for Black, Indigenous, and Multiracial participants these relationships were non-existent. In addition, exploratory analyses revealed lifetime peyote use to confer lowered odds of past month psychological distress for Indigenous populations, and lifetime mescaline use to confer lowered odds of past year suicidal ideation for Multiracial participants.

### Limitations

There are a few limitations that are important to address upfront, many of which have been named in previous population-based survey research on psychedelics^21,27^.

First, and most importantly, the reported associations are correlational and we cannot assume causality of the relationships reported within our study. More explicitly, we cannot infer that MDMA use and psilocybin use decrease distress and suicidality within White participants, nor that these substances fail to do so for Black, Indigenous, and Multiracial participants. Furthermore, the causal interpretations of findings related to MDMA use are limited in particular, as naturalistic samples of MDMA are impure and vary widely in the amount of MDMA they actually contain^28,29^. Additionally, given that the main independent variables are lifetime use (yes/no) variables, we cannot establish clear temporal precedent between substance use and our dependent variables of interest, further weakening causal interpretations of our findings. In addition, the binary nature of our main independent variables precludes us from assessing the frequency, dose, purity, and context of psychedelic use as well.

Next, given the sensitive and stigmatized nature of illegal substance use, underreporting is another limitation to this study, particularly for racial and ethnic minorities who face heightened risk of legal repercussions due to drug use^30^. Underreporting may have lowered the power in our study, making it more difficult to detect significant associations between psychedelic use and distress and suicidality for racial and ethnic minorities.

Lastly, the racial and ethnic groupings included in our study (White, Black, Indigenous, Asian, Multiracial, and Hispanic) are broad, as each of these categories contains many distinct sub-populations that we did not examine in our analyses. Future research should take a more granular approach to studying the intersection between race and ethnicity, psychedelic use, and mental health.

### Potential mechanisms

Even when one takes into account the above limitations, the implications of this study are important. This study demonstrates that race and ethnicity have a significant impact on the associations that psychedelics share with mental health outcomes. Furthermore, although we cannot definitively draw causal conclusions from our study, there are many potential mechanisms to consider that may underlie our observed findings. Although the below reflections remain speculative, they can inform future research on the link between identity, psychedelic use, and mental health.

#### Pre-drug and third-variable factors

It is plausible that third variable factors and pre-drug differences explain the differing associations by race and ethnicity between MDMA and psilocybin use and distress and suicidality. Within population-based survey research on psychedelics, third-variable factors are often cited as a core limitation to the research, as researchers have noted that important pre-drug differences (e.g., greater openness, higher levels of spirituality) that are associated with psychedelic drug use may also confer lowered odds of deleterious mental health outcomes^21,31–33^. In this study, however, the possibility that pre-drug differences explain the differing associations between racial and ethnic groups is a key takeaway and invites further investigation into the characteristics of racial and ethnic minorities who use psychedelic drugs. Furthermore, other population-based survey research has found suggestive evidence that third-variable sociodemographic differences (e.g., socioeconomic status, education) may underlie the associations between naturalistic psychedelic use and lowered odds of mental health disorders as well^34,35^. Therefore, third-variable demographic differences between White and racial and ethnic minority participants may explain why protective associations between psychedelic use and distress and suicidality were found only for White participants. However, at present, there is little descriptive research on racial and ethnic minorities who engage in psychedelic use^19,20^; thus, future investigations into these populations can shed further light on our observed findings.

The study also invites deeper understanding of psychedelic drug use within individuals holding other historically marginalized identities (e.g., low socioeconomic status, sexual/gender minorities, etc.). Similar to the above, there is a dearth of information on the characteristics of individuals who hold diverse identities and engage in psychedelic use, with just a handful of studies exploring this research area. Argento et al. (2017)^36^ conducted a longitudinal study featuring 766 marginalized Canadian women (i.e., women experiencing homelessness, engaging in sex work, HIV+, etc.) and found psychedelic use was associated with significantly decreased risk of suicide. Additionally, a second longitudinal study by Argento et al. (2018)^37^ examined an updated sample from the previous study and found psychedelic use to have a protective moderating effect on the associations between illicit opioid use and suicide risk within an overlapping cohort of 900 marginalized women.

Although these studies make essential contributions to the literature by demonstrating protective effects of psychedelic use in underserved populations, these studies do not examine how specific identity factors might impact the associations between psychedelic use and mental health outcomes. Better understanding how identity may impact the effects of psychedelic substances can serve to improve drug policy, facilitate harm reduction for individuals who use psychedelic substances, and potentially give rise to new sources of support for underserved populations.

#### “Set” and “setting”

To the extent that our findings reflect potential causal links between identity, psychedelic use, and lowered odds of distress and suicidality, “set” and “setting” may explain our results. “Set” (the mindset of an individual taking a psychedelic substance) and the “setting” within which one consumes a psychedelic substance are reported to have a marked impact on the effects of these compounds^38,39^. Unfortunately, in the American “setting,” racism, prejudice, and discrimination are deep-rooted features of the minority experience^40,41^ and may thus negatively impact the minority psychedelic experience. As previously mentioned, due to racism, engaging in illegal psychedelic use can be particularly risky for racial and ethnic minorities; for instance, Black Americans are 5–7 × more likely to be arrested for illegal substance use than are White Americans^30^ despite similar rates of substance use^42^. If one considers the numerous complications that discrimination may introduce into minority “set” and “setting,” it becomes unsurprising that one sees fewer salutary associations for racial and ethnic minorities engaging in naturalistic psychedelic use. Future investigations are needed to examine how prejudice and discrimination may impact the psychedelic experience, an invaluable line of inquiry to create safe and effective psychedelic treatment approaches for minorities.

### Implications for psychedelic-assisted therapy research

The above reflections on minority “set” and “setting” have numerous implications for current psychedelic treatment paradigms as well. Within Western medicine, psychedelic treatment paradigms have mostly been created, assessed, and administered by researchers and therapists of European descent. Thus, current treatment “settings” may not promote salutary outcomes for racial and ethnic minorities and may even be iatrogenic in nature. Additionally, many racial and ethnic minorities may approach psychedelic treatment research with uneasy mind “sets” not only due to the illegal status of psychedelics, but also due to the well-documented history of abuse of minority populations within Western clinical science^43^. Hence, researchers should carefully consider these factors in order to appropriately develop safe and effective psychedelic treatments for racial and ethnic minorities.

Accordingly, researchers should consider conducting trials involving culturally-tailored psychedelic treatment paradigms for minority populations. The handful of protective associations between MDMA use and psilocybin use and distress and suicidality for minority populations within this study warrants further investigation into whether these substances can possibly support salutary mental health outcomes for diverse populations. This notion is further bolstered by the results of our exploratory analyses that revealed mescaline use and peyote use to confer lowered odds of suicidal thinking and psychological distress within Multiracial and Indigenous populations, respectively. The results linking peyote use to lowered odds of distress for Indigenous populations are particularly notable, as peyote has been used by North American Indigenous populations for thousands of years in healing rituals and ceremonies. Hence, these findings invite further inquiry into whether culturally-informed psychedelic therapies, developed in collaboration with the communities they aim to support and in partnership with populations from which many of these substances originate, can provide effective mental health treatments for diverse populations.

Additionally, future research should investigate the generalizability of current findings related to psychedelic-assisted treatments to racial and ethnic minorities. Follow-up studies and secondary data analyses of minority participants in existing psychedelic treatment trials would shed much needed light on the efficacy of these treatments for minority populations, as well as any factors that may bolster or hinder the efficacy of psychedelic treatments for diverse groups.

## Conclusion

MDMA and psilocybin can potentially alleviate many difficult-to-treat mental health disorders. This study demonstrates that race and ethnicity moderate associations between these substances and lowered odds of deleterious mental health outcomes (psychological distress and suicidality) in a cross-sectional survey sample. There is a clear need to better understand psychedelic use in racial and ethnic minority communities, as well as to investigate the therapeutic efficacy of these compounds for minorities. This study brings us closer to harm reduction and more effective drug policy for minorities using psychedelics, as well as closer to ensuring that these potentially salutary compounds can support individuals from all backgrounds.

## Supplementary Information

Supplementary Information.

## Data Availability

The data supporting the findings from this project are publicly available at the Substance Abuse & Mental Health Data Archive (SAMHDA) at the following web address: https://www.datafiles.samhsa.gov/.
