# Supplementary material for: Race and ethnicity moderate the associations between lifetime psychedelic use (MDMA and psilocybin) and psychological distress and suicidality
Source: Sci Rep. 2022 Oct 10;12:16976. doi: 10.1038/s41598-022-18645-3 (PMC9551032; doi:10.1038/s41598-022-18645-3)
Supplement: Supplementary file 1 — Supplementary Information. [file 41598_2022_18645_MOESM1_ESM.docx]

**Supplemental Table 1.** Associations between lifetime LSD, peyote, and mescaline use and past month psychological distress, past year suicidal ideation (S.I), and past year suicidal planning (S.P), by race and ethnicity

|  | | Past Mo. Distress | Past Yr. S.I | Past Yr. S.P |
| --- | --- | --- | --- | --- |
| Group | Lifetime Use | aOR (95% CI)^1^ | aOR (95% CI) | aOR (95% CI) |
| White | LSD | 1.09 (0.99, 1.20) | 1.07 (0.99, 1.16) | 1.08 (0.93, 1.24) |
|  | Peyote | 0.94 (0.82, 1.08) | 0.95 (0.81, 1.10) | 1.06 (0.85, 1.33) |
|  | Mescaline | 0.95 (0.82, 1.10) | 1.01 (0.89, 1.14) | 0.95 (0.73, 1.23) |
| Black | LSD | 0.77 (0.51, 1.15) | 1.20 (0.84, 1.73) | 0.93 (0.55, 1.57) |
|  | Peyote | 1.29 (0.65, 2.54) | 1.21 (0.63, 2.32) | 1.50 (0.52, 4.32) |
|  | Mescaline | 0.98 (0.58, 1.67) | 1.12 (0.60, 2.10) | 1.01 (0.48, 2.11) |
| Indigenous | LSD | 0.63 (0.31, 1.29) | 0.86 (0.44, 1.67) | 0.45 (0.19, 1.09) |
|  | Peyote | **0.55* (0.33, 0.94)** | 1.11 (0.58, 2.13) | 1.54 (0.75, 3.19) |
|  | Mescaline | 1.43 (0.43, 4.75) | 1.23 (0.45, 3.34) | 2.45 (0.59, 10.2) |
| Asian | LSD | 1.25 (0.63, 2.45) | 1.13 (0.56, 2.28) | 1.27 (0.52, 3.06) |
|  | Peyote | 0.47 (0.04, 5.98) | 1.85 (0.30, 11.3) | 9.11 (0.88, 94.8) |
|  | Mescaline | 3.09 (0.62, 15.4) | 0.54 (0.09, 3.39) | 0.07 (0.00, 1.06) |
| Multiracial | LSD | 1.09 (0.72, 1.67) | 0.93 (0.68, 1.26) | 0.89 (0.49, 1.60) |
|  | Peyote | 1.19 (0.69, 2.04) | 1.17 (0.74, 1.83) | 1.37 (0.58, 3.24) |
|  | Mescaline | 0.81 (0.43, 1.54) | **0.57* (0.33, 0.97)** | 1.64 (0.69, 3.87) |
| Hispanic | LSD | 1.13 (0.91, 1.42) | 1.11 (0.90, 1.37) | 0.81 (0.56, 1.18) |
|  | Peyote | 0.94 (0.59, 1.48) | 1.20 (0.78, 1.85) | 0.68 (0.35, 1.35) |
|  | Mescaline | 0.91 (0.61, 1.37) | 1.26 (0.81, 1.97) | 1.28 (0.59, 2.75) |
| ^1^*p<0.05; **p<0.01; ***p<0.001; aOR = adjusted odds ratio; CI = confidence interval | | | | |
|  | | | | |
